# Association of a composite score of relative grip strength and timed up and go test with incident type 2 diabetes mellitus: Guangzhou Biobank Cohort Study

**DOI:** 10.18632/aging.203285

**Published:** 2021-07-16

**Authors:** Xue Liang, Chao Qiang Jiang, Wei Sen Zhang, Feng Zhu, Ya Li Jin, Kar Keung Cheng, Tai Hing Lam, Lin Xu

**Affiliations:** 1 School of Public Health, Sun Yat-Sen University, Guangzhou, China; 2 Guangzhou No.12 Hospital, Guangzhou 510620, China; 3 School of Public Health, The University of Hong Kong, Hong Kong; 4 Institute of Applied Health Research, University of Birmingham, Birmingham, UK

**Keywords:** RGS-TUG score, relative grip strength, timed up and go test, incident type 2 diabetes mellitus

## Abstract

Background: We investigated association of a score incorporating relative grip strength (RGS) and timed up and go (TUG) test with incident type 2 diabetes mellitus (T2DM) in older Chinese.

Methods: Both RGS and TUG scores were classified into tertiles (0~2 points) and summed to yield RGS-TUG score, ranging from 0 to 4 points, with higher points indicating better physical function. Cox proportional hazards regression was used to analyze association of RGS-TUG score with incident T2DM.

Results: 3,892 participants without T2DM were followed up for an average of 3.6 years with 240 developing T2DM. After adjustment, those with the lowest RGS-TUG score, versus the highest, had higher fasting glucose, two-hour post-load glucose and glycosylated hemoglobin A1c, with β (95% confidence interval (CI)) being 0.21 (0.08, 0.33), 1.06 (0.69, 1.43) and 0.16 (0.06, 0.27), respectively. In participants with BMI of ≥25 kg/m2, those with the lowest RGS-TUG score showed a higher risk of T2DM (adjusted hazard ratio 3.01, 95% CI 1.04–8.69). No association was found for BMI of 18.5~<25 kg/m2 (P for interaction < 0.05).

Conclusions: This is the first study showing lower RGS-TUG score was associated with increased glycemia and incident T2DM in older people with overweight/obesity. The underlying mechanisms warrant further investigation.

## INTRODUCTION

China has the largest number of adults with diabetes mellitus (DM) in the world [1]. The prevalence of DM in China increased from 0.67% in 1980 [2] to 10.9% in 2013 [3], then to 12.8% in 2017 [4]. Low muscle strength has been identified as a risk factor for diabetes [5]. Muscle mass and muscle function play important roles in glucose metabolism, and improving muscle strength through resistance training may improve glycemic control in patients with diabetes [6, 7]. In non-trial settings, a commonly used measure of muscle strength is grip strength [8]. However, results of the association between grip strength and incident type 2 diabetes mellitus (T2DM) were inconsistent, with some showing no association [9, 10], but others showing an inverse association [11, 12]. Moreover, no association between grip strength and T2DM was found in a Mendelian randomization study [13], whereas an inverse association was reported in a meta-analysis of 13 cohort studies [5]. Besides grip strength, timed up and go (TUG) test is also used in evaluating physical function, specifically balance and gait in older people [14]. We found only one study examining the association of TUG test with incident T2DM, which showed no association [15].

Grip strength [16] and TUG test [17] represent the upper and lower limb muscle strength, respectively. A composite score including both of these two measures may provide more comprehensive assessment of general muscle strength and physical function. Our PubMed search up to 14 October 2020 using key words of upper limb strength, grip strength, lower limb strength, TUG test and incident T2DM found no article on the association of integrated upper and lower limb muscle strength with the incidence of T2DM. We therefore analyzed grip strength and TUG test with incident T2DM separately, and then derived a composite score based on these two measures and examined whether it predicted incident T2DM prospectively using data from the Guangzhou Biobank Cohort Study (GBCS).

## RESULTS

**Figure 1:**
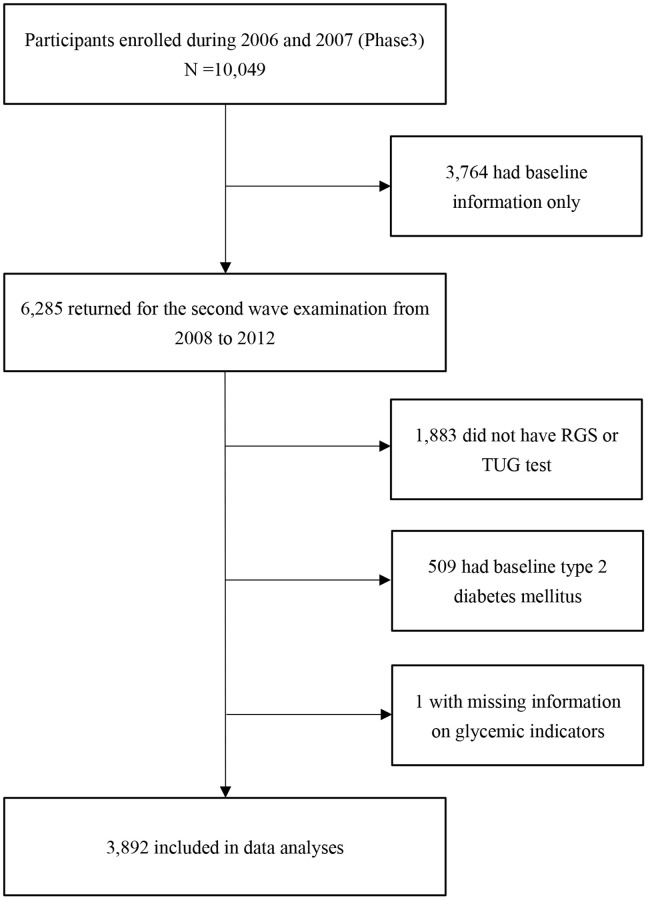
**Flow chart of the study sample selection.** Abbreviations: RGS: relative grip strength; TUG test: timed up and go test.

Figure 1 shows that of 10,049 participants enrolled from 2006 to 2007, 6,285 returned for the second wave examination from 2008 to 2012. After excluding those with missing information on RGS or TUG test (*n* = 1,883), baseline T2DM (*n* = 509) and loss to follow-up for glycemic indicators (*n* = 1), 3,892 were included in the current analyses. Of the 3,892 participants, 583 (15.0%), 915 (23.5%), 1,124 (28.9%), 785 (20.1%) and 485 (12.5%) had RGS-TUG score of 4, 3, 2, 1 and 0 points, respectively. During 13,856 person-years of follow-up (average 3.6 years, standard deviation = 0.7 year), 240 participants developed incident T2DM.

Table 1 shows that participants who had lower RGS-TUG score were older, had fewer men, lower education, and more with manual occupation, more current smokers and never alcohol users (all *P* < 0.001). Lower RGS-TUG score was associated with poorer self-rated health, higher BMI, fasting glucose and 2hPG at baseline (*P* from 0.03 to <0.001). There was no significant difference in physical activity or HbA_1c_ at baseline by RGS-TUG score (*P* = 0.43 and 0.55, respectively).

**Table 1:** Baseline characteristics by physical function, as indicated by RGS-TUG score in 3,892 participants without type 2 diabetes mellitus in the Guangzhou Biobank Cohort Study in 2006–8.

	**RGS-TUG score**					***P* values**
	**4 (best)**	**3**	**2**	**1**	**0 (poorest)**	
Number of participants	583	915	1124	785	485	
Sex, % men	10.4	9.9	11.7	5.3	5.0	<0.001
Age, years, mean (SD)	57.3 (5.5)	57.7 (6.1)	58.7 (6.9)	60.2 (7.8)	65.0 (8.5)	<0.001
Relative grip strength, kg per kg/m^2^, mean (SD)	1.5 (0.3)	1.2 (0.3)	1.0 (0.3)	0.9 (0.2)	0.7 (0.1)	<0.001
TUG test, second, mean (SD)	4.3 (0.3)	4.7 (0.4)	5.0 (0.7)	5.5 (0.8)	6.3 (1.3)	<0.001
Education, %						
Primary or below	19.2	26.5	30.9	45.2	64.4	<0.001
Middle school	72.6	66.2	59.6	48.6	33.0	
College or above	8.2	7.3	9.6	6.1	2.6	
Occupation, %						
Manual	59.5	59.0	64.4	68.8	73.8	<0.001
Non-manual	22.9	21.2	17.6	15.4	10.6	
Others	17.6	19.9	18.0	15.8	15.6	
Smoking status, %						
Never	94.7	92.0	90.4	94.9	91.0	<0.001
Former	2.4	4.3	4.1	3.2	5.0	
Current	2.9	3.8	5.4	1.9	4.0	
Alcohol use, %						
Never	29.4	35.6	32.9	37.9	42.2	<0.001
Former	2.6	4.2	3.5	3.4	4.2	
Current	68.0	60.3	63.7	58.8	53.7	
Physical activity, %						
Inactive	6.4	6.8	7.7	7.5	5.3	0.43
Minimally active	25.3	27.4	24.2	27.4	32.7	
Active	68.4	65.8	68.1	65.1	62.0	
Self-rated health, % poor	16.3	18.3	17.3	20.6	28.2	<0.001
BMI, kg/m^2^, mean (SD)	22.4 (2.8)	23.1 (2.9)	23.5 (3.0)	24.5 (3.2)	25.7 (3.5)	<0.001
Fasting glucose, mmol/l, mean (SD)	5.3 (0.5)	5.3 (0.5)	5.3 (0.5)	5.4 (0.6)	5.4 (0.6)	<0.001
2-hour post-load glucose, mmol/l, mean (SD)	6.8 (1.7)	6.9 (1.4)	7.1 (1.6)	7.2 (1.5)	7.6 (1.8)	0.03
HbA_1c_, %, mean (SD)	5.8 (0.4)	5.8 (0.4)	5.9 (0.4)	5.9 (0.5)	5.9 (0.3)	0.55

RGS-TUG score: a composite score of relative grip strength (RGS) and timed up and go (TUG) test, with 4 indicating best physical function and 0 indicating poorest physical function; Abbreviations: BMI: body mass index; HbA_1c_: hemoglobin A_1c_; SD: standard deviation; TUG: timed up and go test.

Table 2 shows that, of 3,892 participants, 2,472 participants had normal weight, 1,251 had overweight/obesity, and 169 had underweight (BMI of <18.5 kg/m^2^). The mean BMI across five composite scores was from 21.96 to 22.78 kg/m^2^ in participants with normal weight, and from 26.65 to 28.05 kg/m^2^ in those with overweight/obesity. After adjusting for sex, age, education, occupation, smoking status, alcohol use, self-rated health and fasting glucose at baseline, lower RGS-TUG score was associated with higher fasting glucose, 2hPG and HbA_1c_ at follow-up in all participants without baseline T2DM (*P* for trend from 0.02 to <0.001). Those with the lowest, versus the highest, RGS-TUG score had higher fasting glucose, 2hPG and HbA_1c_ at follow-up, with the adjusted β (95% CI) being 0.21 (0.08, 0.33) mmol/l, 1.06 (0.69, 1.43) mmol/l and 0.16 (0.06, 0.27) %, respectively. After similar adjustment, a positive association was observed between tertiles of RGS and glycemic indicators, with β (95% CI) being 0.03 (0.002, 0.18) mmol/l, 0.59 (0.34, 0.85) mmol/l and 0.09 (0.02, 0.16) % for fasting glucose, 2hPG and HbA_1c_, respectively. However, no association between TUG test and glycemic indicators including fasting glucose, 2hPG and HbA_1c_ was found. (Supplementary Table 1).

**Table 2:** Regression coefficients (βs and 95% confidence intervals) for glycemic indicators at follow-up in participants without baseline type 2 diabetes mellitus by baseline RGS-TUG score and obesity status.

		**RGS-TUG score**					***P* for trend**
		**4 (best)**	**3**	**2**	**1**	**0 (poorest)**	
**No. of participants**	Total	583	915	1,124	785	485	
	Underweight	47	49	50	19	4	
	Normal weight	436	645	739	448	204	
	Overweight/obesity	100	221	335	318	277	
**BMI, kg/m^2^, mean (SD)**	Total	22.39 (2.78)	23.05 (2.90)	23.55 (3.04)	24.48 (3.23)	25.74 (3.45)	
	Normal weight	21.96 (1.66)	22.13 (1.67)	22.34 (1.68)	22.56 (1.58)	22.78 (1.56)	
	Overweight/obesity	26.65 (1.33)	26.94 (1.57)	27.13 (1.78)	27.61 (2.09)	28.05 (2.46)	
**Fasting glucose, mmol/l**							
Total	Crude model	0.00	0.06 (–0.03, 0.15)	0.12 (0.04, 0.21)^**^	0.14 (0.05, 0.23)^**^	0.25 (0.15, 0.36)^***^	<0.001
	Adjusted model^†^	0.00	0.04 (–0.06, 0.13)	0.09 (–0.01, 0.18)	0.07 (–0.04, 0.18)	0.21 (0.08, 0.33)^**^	0.004
Normal weight	Crude model	0.00	0.02 (–0.07, 0.10)	0.09 (0.01, 0.17)^*^	0.04 (–0.05, 0.13)	0.14 (0.02, 0.26)^*^	0.02
	Adjusted model^†^	0.00	–0.01 (–0.10, 0.08)	0.04 (–0.04, 0.13)	–0.03 (–0.13, 0.08)	0.08 (–0.06, 0.22)	0.51
Overweight/obesity	Crude model	0.00	0.13 (–0.12, 0.39)	0.10 (–0.14, 0.34)	0.15 (–0.09, 0.39)	0.17 (–0.08, 0.42)	0.23
	Adjusted model^†^	0.00	0.18 (–0.09, 0.46)	0.18 (–0.09, 0.46)	0.19 (–0.10, 0.47)	0.34 (0.03, 0.65)^*^	0.08
**2-hour post-load glucose, mmol/l**							
Total	Crude model	0.00	0.30 (0.04, 0.56)^*^	0.53 (0.28, 0.78)^***^	0.83 (0.57, 1.10)^***^	1.49 (1.19, 1.79)^***^	<0.001
	Adjusted model^†^	0.00	0.17 (–0.10, 0.44)	0.39 (0.12, 0.66)^**^	0.44 (0.13, 0.75)^**^	1.06 (0.69, 1.43)^***^	<0.001
Normal weight	Crude model	0.00	0.12 (–0.13, 0.36)	0.45 (0.21, 0.69)^***^	0.61 (0.34, 0.88)^***^	1.02 (0.67, 1.36)^***^	<0.001
	Adjusted model^†^	0.00	–0.02 (–0.28, 0.23)	0.26 (–0.001, 0.52)	0.21 (–0.10, 0.51)	0.61 (0.18, 1.03)^**^	0.003
Overweight/obesity	Crude model	0.00	0.65 (–0.06, 1.36)	0.40 (–0.28, 1.07)	0.75 (0.07, 1.42)^*^	1.32 (0.63, 2.02)^***^	<0.001
	Adjusted model^†^	0.00	0.73 (–0.04, 1.51)	0.59 (–0.18, 1.36)	0.59 (–0.22, 1.40)	1.12 (0.24, 1.99)^*^	0.07
**HbA_1c_, %**							
Total	Crude model	0.00	0.09 (0.02, 0.16)^*^	0.08 (0.01, 0.14)^*^	0.14 (0.07, 0.22)^***^	0.22 (0.14, 0.31)^***^	<0.001
	Adjusted model^†^	0.00	0.05 (–0.03, 0.12)	0.05 (–0.03, 0.13)	0.06 (–0.03, 0.14)	0.16 (0.06, 0.27)^**^	0.02
Normal weight	Crude model	0.00	0.05 (–0.02, 0.12)	0.05 (–0.02, 0.12)	0.06 (–0.01, 0.14)	0.11 (0.005, 0.21)^*^	0.03
	Adjusted model^†^	0.00	0.01 (–0.07, 0.09)	0.02 (–0.06, 0.10)	–0.01 (–0.10, 0.09)	0.08 (–0.05, 0.21)	0.57
Overweight/obesity	Crude model	0.00	0.20 (0.02, 0.39)^*^	0.10 (–0.08, 0.27)	0.20 (0.03, 0.38)^*^	0.26 (0.07, 0.44)^**^	0.02
	Adjusted model^†^	0.00	0.17 (–0.04, 0.37)	0.11 (–0.09, 0.32)	0.13 (–0.09, 0.34)	0.20 (–0.04, 0.44)	0.29

RGS-TUG score: a composite score of relative grip strength and timed up and go test, with 4 indicating best physical function and 0 indicating poorest physical function; Underweight: BMI <18.5 kg/m^2^; Normal weight: 18.5 kg/m^2^ ≤ BMI <25 kg/m^2^; Overweight/obesity: BMI ≥25 kg/m^2^; Abbreviations: BMI: body mass index; SD: standard deviation; HbA_1c_: glycosylated hemoglobin A_1c_.

^†^Adjusting for sex, age, education, occupation, smoking status, alcohol use, self-rated health and fasting glucose at baseline.

^*^P < 0.05; ^**^P < 0.01; ^***^P < 0.001.

**Figure 2:**
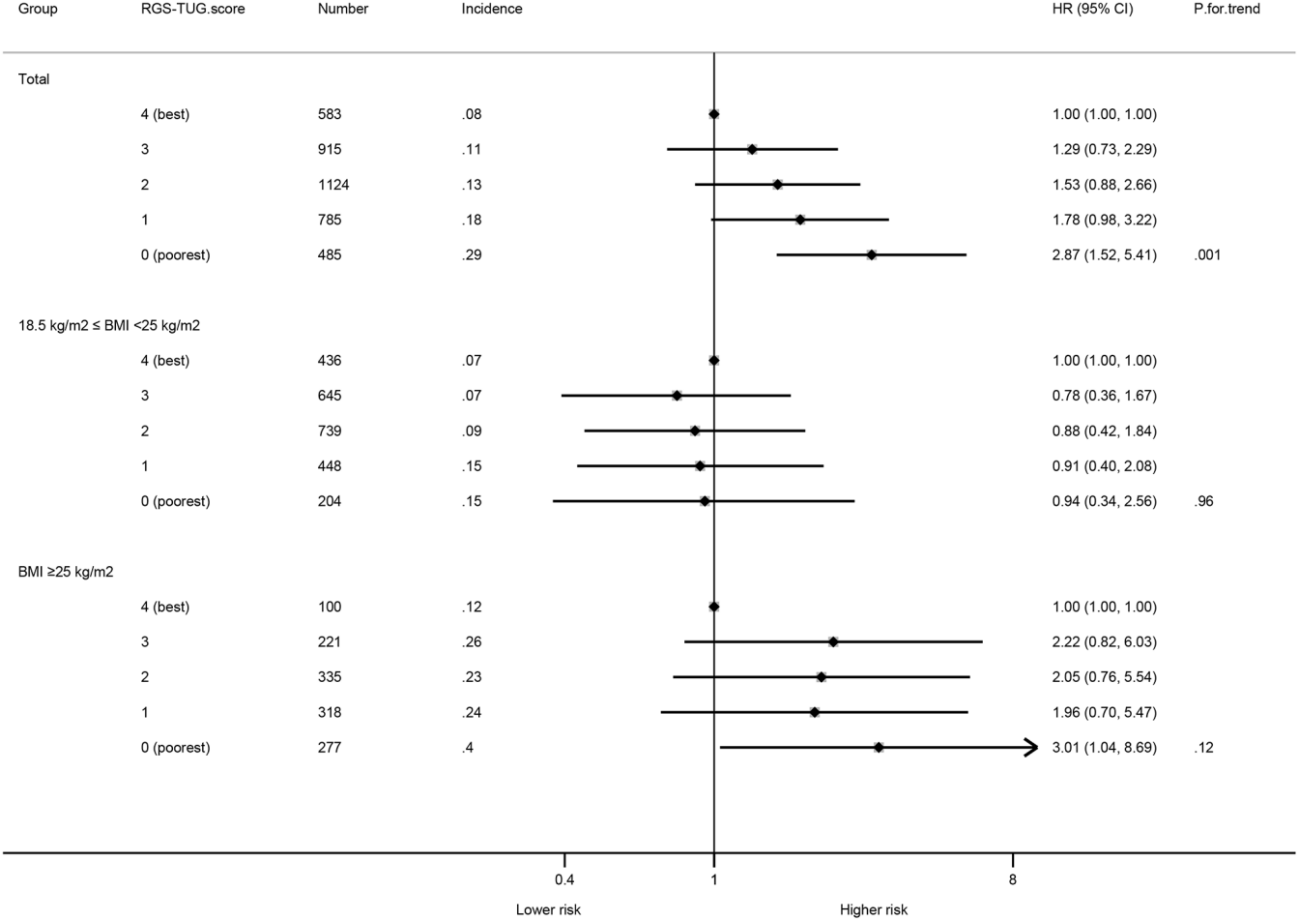
**Associations between RGS-TUG score and incident type 2 diabetes mellitus.** The HRs and 95%CIs above were adjusted for sex, age, education, occupation, smoking status, alcohol use and self-rated health. Abbreviations: Number: number of participants; Incidence: Incidence of T2DM per 100 person-year.

Table 3 shows that lower RGS-TUG score was associated with a higher incidence of T2DM in the fully adjusted model (*P* for trend = 0.001). In participants with BMI ≥25 kg/m^2^, compared with those with the highest RGS-TUG score, the adjusted HR (95% CI) for incident T2DM in those with the lowest RGS-TUG score was 3.01 (1.04, 8.69) but no association was found in participants with a BMI of 18.5~<25 kg/m^2^ (HR 0.94, 95% CI 0.34, 2.56). The association remained and appeared to be weaker in total participants (HR 2.87, 95% CI 1.52, 5.41) (Figure 2). Similar associations were also found in the analyses of RGS (Supplementary Table 2). Compared to participants with the highest RGS, those in the 2nd and the lowest tertile of RGS showed higher risk of incident T2DM (HR (95% CI) 1.96 (1.26, 3.07) and 2.52 (1.58, 4.04), respectively), but no association after stratifying by BMI groups (Supplementary Figure 1). No significant association of TUG test with incident T2DM was found in total participants or by BMI groups (Supplementary Figure 2). Results of the measures comparing fitness or distinguishing ability of the models using the composite score, TUG test and RGS, separately, were shown in the Supplementary Table 3. For model using composite score, TUG or RGS separately, the C-index (95% CI) was 0.60 (0.56, 0.64), 0.58 (0.54, 0.61) and 0.56 (0.53, 0.58), CPE (95% CI) was 0.61 (0.58, 0.64), 0.59 (0.55, 0.62) and 0.58 (0.54, 0.61), AUC (95% CI) was 0.61 (0.58, 0.65), 0.58 (0.55, 0.62) and 0.58 (0.55, 0.61), AIC was 3982.21, 3994.56 and 6169.54 and –2 Log likelihood was 3980.21, 3990.56 and 6165.54, respectively.

**Table 3:** Crude and adjusted hazards ratios (95% confidence intervals) for incident type 2 diabetes mellitus (T2DM) during the follow-up from March 2008 to December 2012 by baseline RGS-TUG score and stratified by obesity status.

	**Number**	**Incidence of T2DM per 100 person-year**	**Crude model HR (95% CI)**	**Adjusted model^†^ HR (95% CI)**
**Total**				
RGS-TUG score				
4 (best)	583	0.08	0.00	0.00
3	915	0.11	1.42 (0.82, 2.44)	1.29 (0.73, 2.29)
2	1,124	0.13	1.68 (1.00, 2.81)^*^	1.53 (0.88, 2.66)
1	785	0.18	2.43 (1.45, 4.07)^**^	1.78 (0.98, 3.22)
0 (poorest)	485	0.29	3.76 (2.24, 6.32)^***^	2.87 (1.52, 5.41)^**^
*P* for trend			<0.001	0.001
**Normal weight**				
RGS-TUG score				
4 (best)	436	0.07	0.00	0.00
3	645	0.07	1.07 (0.52, 2.21)	0.78 (0.36, 1.67)
2	739	0.09	1.38 (0.70, 2.72)	0.88 (0.42, 1.84)
1	448	0.15	2.39 (1.22, 4.68)^*^	0.91 (0.40, 2.08)
0 (poorest)	204	0.15	2.37 (1.08, 5.18)^*^	0.94 (0.34, 2.56)
*P* for trend			0.001	0.96
**Overweight/obesity**				
RGS-TUG score				
4 (best)	100	0.12	0.00	0.00
3	221	0.26	2.14 (0.81, 5.64)	2.22 (0.82, 6.03)
2	335	0.23	1.95 (0.76, 5.00)	2.05 (0.76, 5.54)
1	318	0.24	2.06 (0.80, 5.29)	1.96 (0.70, 5.47)
0 (poorest)	277	0.40	3.35 (1.33, 8.44)^*^	3.01 (1.04, 8.69)^*^
*P* for trend			0.006	0.12

RGS-TUG score: a composite score of relative grip strength (RGS) and timed up and go (TUG) test, with 4 indicating best physical function and 0 indicating poorest physical function.

Incident T2DM: defined by a history of self-reported physician-diagnosed diabetes or glucose-lowering treatment during follow up or fasting glucose ≥7.0 mmol/l or 2hPG ≥11.1 mmol/l and without T2DM at baseline; Normal weight: 18.5 kg/m^2^ ≤ BMI <25 kg/m^2^; Overweight/obesity: BMI ≥25 kg/m^2^.

^†^adjusting for sex, age, education, occupation, smoking status, alcohol use and self-rated health.

^*^P < 0.05; ^**^P < 0.01; ^***^P < 0.001.

## DISCUSSION

Our study is the first to show an inverse association of general physical function, measured by a composite score integrating RGS and TUG test, namely, the RGS-TUG score, with the incidence of T2DM. Poorer physical function was associated with a two-fold higher risk of incident T2DM only in participants with higher BMI. No association was found in those with a BMI of ≥18.5 kg/m^2^ and <25 kg/m^2^. Lower RGS-TUG score was also significantly associated with higher fasting glucose, 2hPG and HbA_1c_, suggesting that RGS-TUG score is a useful indicator of physical function in predicting the risk of incident T2DM in those with BMI ≥25 kg/m^2^.

As a measure of upper limb muscle strength, RGS was associated with the development of incident T2DM. A meta-analysis of 13 cohort studies showed that one SD greater muscle strength, as indicated by grip strength or multiple muscle groups, was significantly associated with a 13% lower risk of incident T2DM [5], which was supported by a Mendelian randomization (MR) study using genetic variants from GWAS of the UK Biobank as instrumental variables [18]. However, another MR study showed no association between grip strength and T2DM, which could be due to the small number of SNPs used (i.e., 2 SNPs only) and limited F-statistic value [13]. Furthermore, non-significant associations of TUG test with incident T2DM and glycemic indicators were found in our study, which was consistent with one prospective cohort study of 1,075 participants [15], although participants who had higher BMI and TUG test (indicating slow gait speed) appeared to have a higher risk of incident diabetes (HR = 1.40, 95% CI 0.81–2.41) in our study. According to this prospective cohort study, participants with a TUG score in the bottom 80% were defined as normal TUG test [15], which equals to a gait speed of 2.18 seconds per meter (s/m). In our study, the gait speed was 1.90 s/m. As the participants in our study were slightly younger than this previous study (59.3 ± 7.3 versus 67.4 ± 5.4 years) [15], the TUG test score was comparable to previous studies. Future larger cohort studies with longer follow-up are needed to fully elucidate the association between gait speed and risk of diabetes.

Previous studies examining the modification effect of BMI on the association of grip strength and diabetes showed inconsistent results [19–22]. One cross-sectional study on 5,039 Japanese men showed that two SD increase in grip strength was associated with 0.64-fold (95% CI 0.49, 0.83) odds of T2DM in participants with BMI >25 kg/m^2^, and the association attenuated to non-significant in those with normal weight (OR 0.79, 95% CI 0.60, 1.06) [19]. Moreover, a cohort study on 5,953 participants from the UK also showed an inverse association of grip strength with incident T2DM in participants with a BMI ≥30 kg/m^2^, but the association was less clear in non-obese participants (HR 4.93 versus 1.51 in the lowest compared with the highest tertile of grip strength) [20]. Another cohort study of 1.5 million Swedish male military conscripts showed that lower muscle strength, measured by the weighted sum of maximal knee extension (weighted × 1.3), elbow flexion (weighted × 0.8), and hand grip (weighted × 1.7), using standard well-validated isometric dynamometer tests, was associated with higher risk of T2DM, and the association did not vary by BMI at baseline [21]. Another cohort study of 394 Japanese-American participants showed that higher grip strength was significantly associated with lower risk of T2DM in those whose BMI was in the lowest quartile, whereas no association was found in those with BMI greater than the 75th percentile [22]. These discrepancies might be due to the heterogeneity in the study samples and methods. Notably, in the Swedish military conscripts study, participants were men aged 18 years and enrolled from 1969 to 1997 [21], who should be healthier and have generally lower BMI than participants of ours and other studies [19, 20]. Moreover, some important confounders such as smoking status and alcohol use [23] were not adjusted [22]. Thus, our results by accounting for a comprehensive set of confounding factors support an inverse association between physical function and incident T2DM risk in older people with higher BMI, highlights the needs for more clinical and public health attentions, and further research on the mechanisms. Lean participants were found to have a delay in the onset of insulin action [24] and lower muscle strength, which may explain the null association in those with normal weight. Furthermore, the diverse association may be due to the different macrophages phenotypes, i.e., M1 macrophages in obese adipose tissue releasing pro-inflammatory cytokines, whereas M2 macrophages in lean adipose tissue releasing anti-inflammatory cytokines [25]. However, the exact mechanisms need to be further investigated.

Some possible explanations for the inverse association between physical function and incident T2DM have been proposed. First, lower muscle strength had an adverse effect on glucose metabolism including increased insulin resistance and pancreatic β-cell dysfunction [26]. Second, strength training was associated with enhanced glucose metabolism through increasing insulin receptor expression [27] and glucose transporter type 4 protein content [27, 28]. Third, individuals with low muscle strength had higher levels of inflammatory cytokines such as interleukin-6 [29] and tumor necrosis factor-alpha [30], which may lead to a higher risk of T2DM. Finally, in the elderly, slow gait speed was an important indicator of frailty [31, 32], which would accelerate the incidence of T2DM [33, 34] due to higher oxidative stress state [35] and shorter telomere length [36].

Strengths of this study included the prospective design, standardized and comprehensive measurement of glycemic indicators (fasting plasma glucose, 2hPG and HbA_1c_), grip strength and gait speed, and adjustment of multiple potential confounders. Moreover, we firstly integrated RGS and TUG test as a composite measure of general physical function, which reflects both upper and lower limb muscle strength. The model using the composite score showed better performance than each score individually. Thus, it may be more comprehensive and informative. However, our study had several limitations. First, residual confounding could not be ruled out. However, we adjusted for most confounders reported in previous papers and additionally adjusted for self-rated health. Second, as RGS and TUG test were performed at baseline, data was only available at one time point and the changes during follow-up were not analyzed. Whether lower muscle metabolism may correlate with decreased glucose metabolism/clearance predisposing to the development of T2DM is unclear [37]. Third, 97% of our participants had BMI of < 30 kg/m^2^, which though obese, were leaner comparing to Western countries [38], and even our obese participants had lower BMI than those in the USA [39]. Hence, our results might not be applicable to gross obesity which is more common in the West. Fourth, as we found evidence that the association of the composite score with incident T2DM varied by adiposity, subgroup analyses were conducted to evaluate the effect modification. However, the exact mechanisms underlying the higher risk of incident T2DM related to lower physical function in older people with overweight/obesity warrants further research. Finally, all participants in the present study were older people with generally lower grip strength and poorer TUG test, which was quite similar to the elderly in China with normal body fat and muscle [15, 40]. Moreover, grip strength of a given age varies by ethnicity, i.e., Chinese had higher grip strength than Africans but lower than Europeans [41]. Thus, our results might not be directly generalizable to other populations.

In conclusion, lower RGS-TUG score was prospectively associated with an increase in glycemia and risk of T2DM in older people with overweight/obesity. The underlying mechanisms warrant further investigation.

## METHODS

### Study sample

The Guangzhou Biobank Cohort Study (GBCS) is an on-going three-way collaborative prospective cohort study among the Guangzhou 12th Hospital, China and the Universities of Hong Kong, China and Birmingham, United Kingdom. Details of the GBCS have been described previously [42]. Briefly, recruitment of participants was from a community social and welfare organization, the Guangzhou Health and Happiness Association for the Respectable Elders (GHHARE). Membership is open to permanent residents aged 50 years or above in Guangzhou with a nominal fee of 4 CNY (≈50 US cents) per month. Baseline information was conducted by face-to-face interviews using a computer-based questionnaire by trained nurses on demographic characteristics, lifestyle, and personal and family medical history. The Guangzhou Medical Ethics Committee of the Chinese Medical Association approved the study, and all participants provided written informed consent before participation.

### Exposure

Relative grip strength (RGS) and timed up and go (TUG) test were examined at baseline and results of those were combined to create a composite exposure RGS-TUG score. Grip strength was assessed using a Jamar Hydraulic Hand Dynamometer two times for each hand, and the maximal value of the average grip strength in left and right hands was used as the absolute grip strength. Grip strength measured by Jamar dynamometer showed good to excellent test-retest reproducibility (*r* > 0.80) [43] and excellent (*r* = 0.98) inter-rater reliability [44]. RGS was calculated by absolute grip strength in kilogram divided by body mass index (BMI, kg/m^2^), expressed as kg per kg/m^2^. RGS was classified into tertiles as follows: tertile 1 (RGS <0.87 kg per kg/m^2^, point = 0); tertile 2 (RGS 0.87–1.15 kg per kg/m^2^, point = 1) and tertile 3 (RGS >1.15 kg per kg/m^2^, point = 2).

TUG test was conducted by asking participants to get up from a chair, walk 2.5 meters around a marker, and return. Nurses recorded the time taken for the test for each participant. The test was performed twice and the scores (in seconds) were averaged. TUG test scores were also categorized into tertiles, i.e., tertile 1 (TUG test <4.7 sec, point = 2); tertile 2 (TUG test 4.7-5.3sec, point = 1) and tertile 3 (TUG test >5.3 sec, point = 0). RGS-TUG score was calculated as the sum of the points of RGS and TUG scores, and categorized into 4, 3, 2, 1 and 0 points, with 4 points indicating best physical function and 0 point indicating poorest physical function.

### Outcomes

The primary outcome was incident T2DM. Other outcomes were glycemic indicators including fasting glucose, two-hour post-load glucose (2hPG) and glycosylated hemoglobin A_1c_ (HbA_1c_) measured at the follow-up examination. Fasting glucose was measured by Shimadzu CL-8000 Clinical Chemistry Analyzer (Shimadzu, Kyoto, Japan). 2hPG was measured 2 hours after 75-gram oral glucose administration in all participants except those with self-reported physician diagnosis of diabetes or with glucose-lowering treatment. T2DM was defined by fasting glucose ≥7.0 mmol/l, and/or 2hPG ≥11.1 mmol/l, or a history of self-reported physician-diagnosed diabetes or glucose-lowering treatment during follow up [45].

### Potential confounders

As sex [46], age [47], lifestyle factors (smoking status and alcohol use [23]) and self-rated health status were associated with RGS, TUG test and T2DM, these factors were considered as potential confounders and adjusted in the regression models. Furthermore, we also included education [48] and occupation to partly account for confounding due to socioeconomic position.

### Statistical analyses

Chi-square tests were used to compare baseline categorical variables by RGS-TUG score, and one-way analyses of variance (ANOVA) for continuous variables. General linear models were used to assess the associations of RGS-TUG score with fasting glucose, 2hPG and HbA_1c_ at follow-up in participants without baseline T2DM, giving regression coefficients (βs) and 95% confidence intervals (CIs). Cox proportional hazards regression was used to assess the association of baseline RGS-TUG score with risk of incident T2DM, giving hazards ratios (HRs) and 95% CIs. Schoenfeld’s residuals were used to test the proportional hazards assumption and if assumption was not satisfied, log-time parametric estimation schemes were used. Harrell’s concordance index (C-index) [49], Gönen and Heller’s Concordance Probability Estimate (CPE), Area Under Curve (AUC), Akaike Information Criterion (AIC) and –2 Log likelihood were used to compare fitness or discrimination ability of the models using the composite score, TUG test and RGS, separately. All participants were followed up from baseline examination to occurrence of T2DM or to the date of repeated examination, whichever date came first. For those who were newly diagnosed as T2DM at the follow-up examination, the censoring date was defined as the midpoint between the baseline and follow-up examinations. Moreover, we also tested for interactions of RGS-TUG score with sex, age (<70 and ≥70 years) and BMI groups (BMI <25 and ≥25 kg/m^2^). As a significant interaction between RGS-TUG score and BMI groups was found (*P* for interaction: <0.001 for incident T2DM and from 0.002 to <0.001 for glycemic indicators), subgroup analyses stratifying by BMI groups were conducted. No interaction of RGS-TUG score with sex and age groups was found (*P* for interaction = 0.95 and 0.94 for incident T2DM, respectively). Furthermore, analyses of RGS and TUG test with glycemic indicators and incident T2DM were also conducted separately. Effect modification was found in BMI groups with RGS (*P* for interaction <0.001) but not with TUG test (*P* for interaction = 0.28). Associations of RGS and TUG test with incident T2DM did not vary by sex (*P* for interaction = 0.62 and 0.34, respectively) and age groups (both *P* for interaction = 0.57). Statistical analyses were done using Stata version 16.0 (STATA Corp LP, TX, USA) and R program version 4.0.2 (MO, USA). All tests were two-sided with *P* < 0.05 as statistically significant.

## Supplementary Materials

Supplementary Figures

Supplementary Tables
