# Supplementary material for: Association of a composite score of relative grip strength and timed up and go test with incident type 2 diabetes mellitus: Guangzhou Biobank Cohort Study
Source: Aging (Albany NY). 2021 Jul 16;13(14):18376–91. doi: 10.18632/aging.203285 (PMC8351683; doi:10.18632/aging.203285)
Supplement: Supplementary Figures [file aging-13-203285-s001.pdf]

SUPPLEMENTARY FIGURES

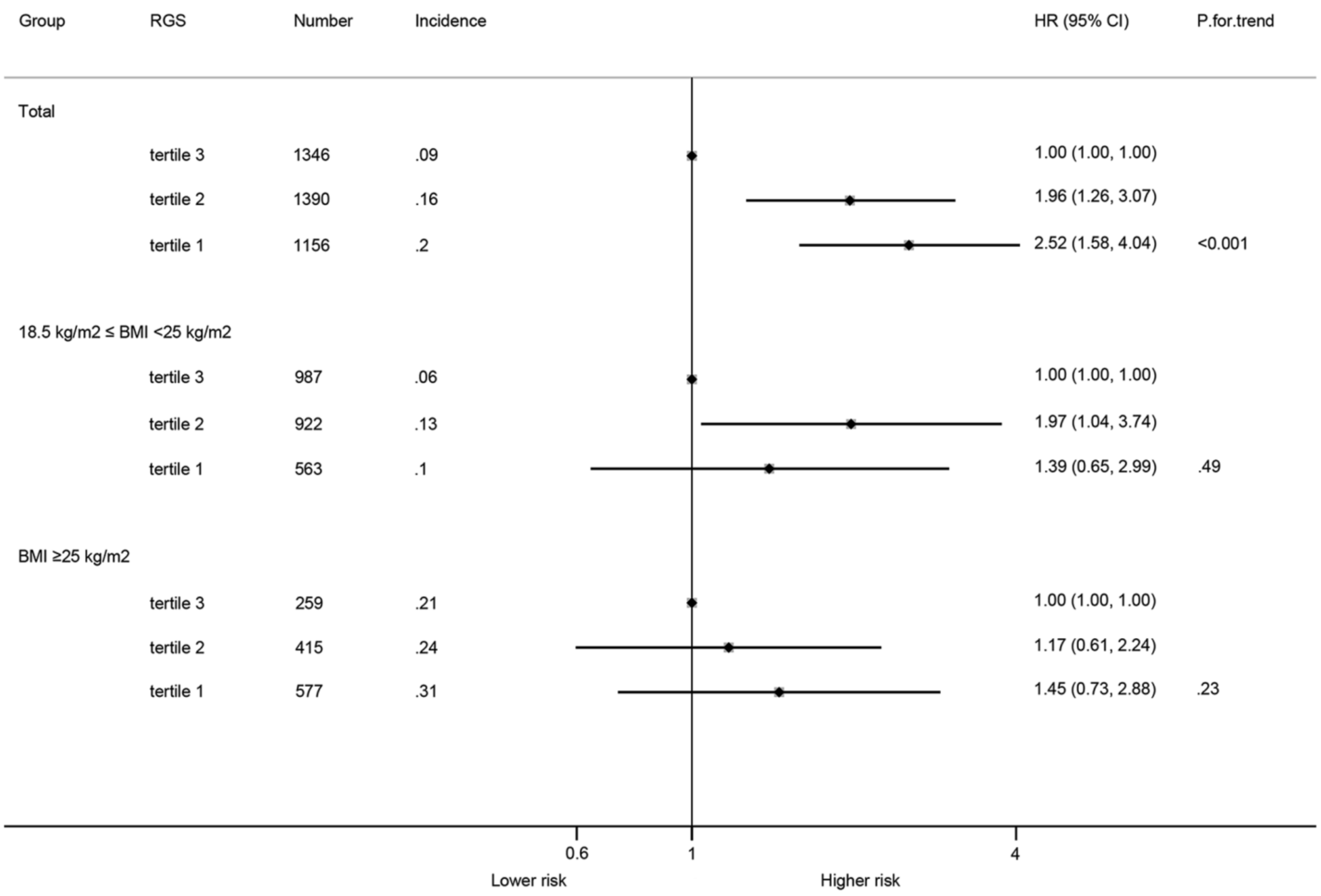

Supplementary Figure 1. Associations between RGS and incident type 2 diabetes mellitus. The HRs and 95%CIs above were adjusted for sex, age, education, occupation, smoking status, alcohol use and self-rated health. Abbreviations: RGS: relative grip strength; Number: number of participants; Incidence: Incidence of T2DM per 100 person-year.

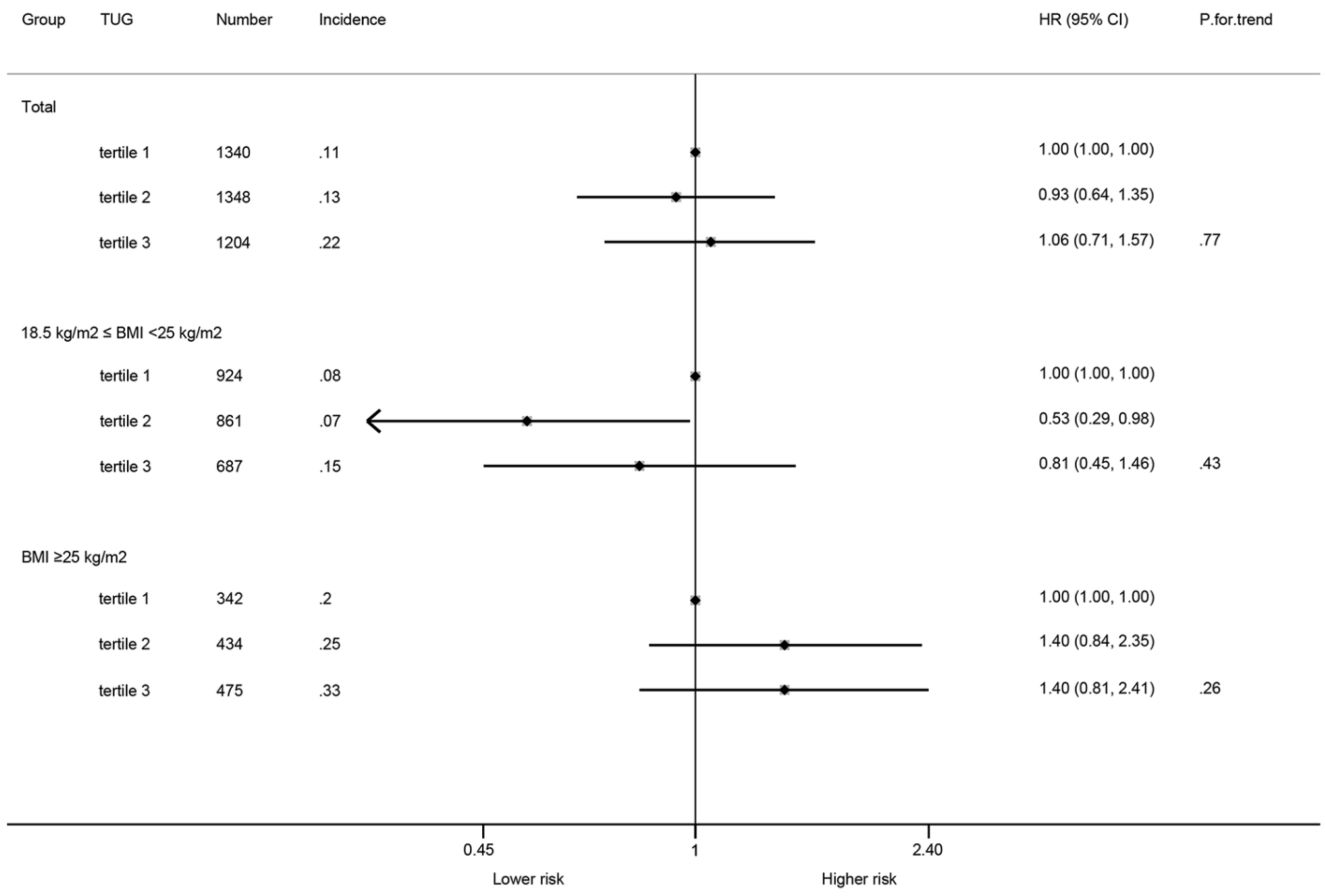

Supplementary Figure 2. Associations between TUG test and incident type 2 diabetes mellitus. The HRs and 95%CIs above were adjusted for sex, age, education, occupation, alcohol use, self-rated health and body mass index. Abbreviations: TUG test: timed up and go test; Number: number of participants; Incidence: Incidence of T2DM per 100 person-year.
