# Supplementary material for: Association of a composite score of relative grip strength and timed up and go test with incident type 2 diabetes mellitus: Guangzhou Biobank Cohort Study
Source: Aging (Albany NY). 2021 Jul 16;13(14):18376–91. doi: 10.18632/aging.203285 (PMC8351683; doi:10.18632/aging.203285)
Supplement: Supplementary Tables [file aging-13-203285-s002.pdf]

SUPPLEMENTARY TABLES

Supplementary Table 1. Regression coefficients (βs and 95% confidence intervals) for glycemic indicators at follow-up by baseline groups of relative grip strength and timed up and go test in participants without baseline type 2 diabetes mellitus.

	Fasting glucose, mmol/l		2-hour post-load glucose, mmol/l		Hemoglobin A_{1c}, %	
	**Crude model**	**Adjusted model†**	**Crude model**	**Adjusted model†**	**Crude model**	**Adjusted model†**
**Tertiles of relative grip strength (RGS), kg per kg/m²**						
3rd (*n* = 1,346)	0.00	0.00	0.00	0.00	0.00	0.00
2nd (*n* = 1,390)	0.07 (0.002, 0.13)*	0.03 (–0.05, 0.11)	0.46 (0.27, 1.07)***	0.33 (0.09, 0.56)**	0.07 (0.02, 0.12)**	0.04 (–0.02, 0.11)
1st (*n* = 1,156)	0.15 (0.08, 0.21)***	0.03 (0.002, 0.18)*	0.88 (0.68, 1.07)***	0.59 (0.34, 0.85)***	0.13 (0.07, 0.18)***	0.09 (0.02, 0.16)*
P for trend	<0.001	0.04	<0.001	<0.001	<0.001	0.02
**Tertiles of timed up and go (TUG) test, second**						
1st (*n* = 1,340)	0.00	0.00	0.00	0.00	0.00	0.00
2nd (*n* = 1,348)	0.07 (0.01, 0.14)*	0.04 (–0.03, 0.11)	0.16 (–0.03, 0.35)	0.01 (–0.19, 0.21)	0.04 (–0.01, 0.09)	0.01 (–0.05, 0.06)
3rd (*n* = 1,204)	0.12 (0.05, 0.18)**	0.05 (–0.03, 0.13)	0.73 (0.54, 0.93)***	0.23 (–0.0001, 0.45)	0.09 (0.04, 0.15)**	0.01 (–0.06, 0.07)
P for trend	0.001	0.18	<0.001	0.06	0.001	0.79

†Adjusting for sex, age, education, occupation, smoking status (only for analysis of RGS), alcohol use, self-rated health, body mass index (only for analysis of TUG), and fasting glucose at baseline, as appropriate.
*$P < 0.05$; **$P < 0.01$; ***$P < 0.001$.

Supplementary Table 2. Crude and adjusted hazards ratios (95% confidence intervals) for incident type 2 diabetes mellitus (T2DM) during the follow-up from March 2008 to December 2012 by baseline groups of relative grip strength and timed up and go test.

	Number	Incidence of T2DM per 100 person-year	Crude model	Adjusted model†
**Tertiles of relative grip strength (RGS), kg per kg/m^2**				
**Total**				
3rd	1,346	0.09	0.00	0.00
2nd	1,390	0.16	1.77 (1.26, 2.49)**	1.96 (1.26, 3.07)**
1st	1,156	0.20	2.26 (1.61, 3.17)***	2.52 (1.58, 4.04)***
P for trend			<0.001	<0.001
**Normal weight**				
3rd	987	0.06	0.00	0.00
2nd	922	0.13	2.01 (1.26, 3.21)**	1.97 (1.04, 3.74)*
1st	563	0.10	1.57 (0.91, 2.72)	1.39 (0.65, 2.99)
P for trend			0.06	0.49
**Overweight/obesity**				
3rd	259	0.21	0.00	0.00
2nd	415	0.24	1.17 (0.69, 1.96)	1.17 (0.61, 2.24)
1st	577	0.31	1.51 (0.94, 2.43)	1.45 (0.73, 2.88)
P for trend			0.06	0.23
**Tertiles of timed up and go (TUG) test, second**				
1st	1,340	0.11	0.00	0.00
2nd	1,348	0.13	1.18 (0.84, 1.67)	0.93 (0.64, 1.35)
3rd	1,204	0.22	2.07 (1.51, 2.83)***	1.06 (0.71, 1.57)
P for trend			<0.001	0.77

Incident T2DM: defined by a history of self-reported physician-diagnosed diabetes or glucose-lowering treatment during follow up or fasting glucose ≥7.0 mmol/l or 2hPG ≥11.1 mmol/l and without T2DM at baseline; Normal weight: 18.5 kg/m^2 ≤ BMI <25 kg/m^2; Overweight/obesity: BMI ≥25 kg/m^2.

†Adjusting for sex, age, education, occupation, smoking status (only for analysis of RGS), alcohol use, self-rated health and body mass index (only for analysis of TUG).

**P < 0.01; ***P < 0.001.

Supplementary Table 3. Comparisons of the Cox proportional hazards regression models for incident diabetes using the RGS-TUG score, relative grip strength (RGS) and timed up and go (TUG) test, separately.

	RGS-TUG score	RGS	TUG test
C-index (95% CI)	0.60 (0.56, 0.64)	0.58 (0.54, 0.61)	0.56 (0.53, 0.58)
CPE (95% CI)	0.61 (0.58, 0.64)	0.59 (0.55, 0.62)	0.58 (0.54, 0.61)
AUC (95% CI)	0.61 (0.58, 0.65)	0.58 (0.55, 0.62)	0.58 (0.55, 0.61)
AIC	3982.21	3994.56	6169.54
–2 Log likelihood	3980.21	3990.56	6165.54

Abbreviations: CI: confidence interval; C-index: Harrell's concordance index; CPE: Gönen and Heller's Concordance Probability Estimate; AUC: Area Under Curve; AIC: Akaike Information Criterion.
